# Association of thymidylate synthase gene 3'-untranslated region polymorphism with sensitivity of non-small cell lung cancer to pemetrexed treatment: TS gene polymorphism and pemetrexed sensitivity in NSCLC

**DOI:** 10.1186/1423-0127-20-5

**Published:** 2013-01-25

**Authors:** Xia Wang, Yadi Wang, Yue Wang, Jian Cheng, Yanyun Wang, Minwen Ha

**Affiliations:** 1 Department of Oncology, First Affiliated Hospital of Liaoning Medical College, Jinzhou, China; 2 Department of Oncology, Third Affiliated Hospital of Liaoning Medical College, Jinzhou, China

**Keywords:** Lung adenocarcinoma, Non-small cell lung cancer, Pemetrexed treatment, Thymidylate synthase, *TS* gene polymorphism

## Abstract

**Background:**

Thymidylate synthase (TS) is a key enzyme responsible for DNA synthesis and repair. Altered expression of TS protein or *TS* gene polymorphisms has been associated with cancer progression and treatment response. This study investigated the expressions of TS and its gene SNPs in non-small cell lung cancer (NSCLC), and then its association with sensitivity to pemetrexed treatment. Immunohistochemistry and qRT-PCR were performed on 160 resected NSCLC specimens and corresponding normal tissues to assess the expressions of TS protein and *TS* mRNA, and for associations with clinicopathological data. Blood samples of 106 lung adenocarcinoma patients were examined for polymorphisms of the *TS* gene 3’-UTR 1494del 6 bp, which was then investigated for associations with responses of the patients to pemetrexed treatment and survival.

**Results:**

Expression of both TS protein and its mRNA was elevated in NSCLC tissues compared with matched normal tissues, and significantly higher in lung squamous cell carcinoma than in lung adenocarcinoma. TS expression was associated with poor tumor differentiation. Furthermore, the genotyping data showed that 56% of lung adenocarcinoma patients had the *TS* gene 3’-UTR 1494 bp (−6 bp/-6 bp) genotype and the rest had *TS* gene 3’-UTR 1494 bp (−6 bp/+6 bp). There was no *TS* 3’-UTR 1494 bp (+6 bp/+6 bp) genotype in any patients. Statistical analysis revealed that gender, tumor stage, and *TS* 3’-UTR 1494del 6 bp polymorphism were significant prognostic factors after short-term pemetrexed treatment. Log-rank analysis revealed that patients with the (−6 bp/-6 bp) genotype had significantly better progression-free and overall survival than patients with (−6 bp/+6 bp).

**Conclusions:**

This study showed that TS protein is highly expressed in NSCLC and that polymorphisms of *TS* 3’-UTR 1494del 6 bp are associated with sensitivity of lung adenocarcinoma patients to pemetrexed treatment. This suggests that *TS* gene polymorphisms should be further evaluated as prognostic markers for personalized therapy in lung adenocarcinoma.

## Background

Lung cancer was the leading cause of cancer death in men and the second leading cause in women worldwide in 2008. Although lung cancer incidence is slowly decreasing in developed countries, the number of lung cancer cases is still increasing in China and other developing countries [1]. Over 75% of all lung cancer cases are NSCLC, which include adenocarcinoma, squamous cell carcinoma, and large-cell carcinoma [2]. Common treatments for NSCLC include surgery, chemotherapy, radiation therapy, and palliative care depending on the cancer type, stage, and other factors. Primary chemotherapy is also given for advanced and metastatic NSCLC. A combination of platinum chemotherapy compounds (cisplatin or carboplatin) with gemcitabine, vinorelbine, or taxanes (paclitaxel or docetaxel) is a widely used regimen for first-line treatment of stage IIIB or IV NSCLC [2]. However, there is no standard regimen for local recurrence of NSCLC. In 2006, the National Comprehensive Cancer Network recommended pemetrexed as a single agent for second-line treatment of advanced NSCLC [3].

Pemetrexed is an antifolate drug that inhibits growth of a variety of tumor types by targeting the folate-dependent enzymes dihydrofolate reductase, glycinamide ribonucleotide formyltransferase, and thymidylate synthase (TS); but is most potent on TS [4,5]. To date, three large phase III clinical trials have provided strong evidence for use of pemetrexed in control of NSCLC [5-8]. The data also showed that tumor histology was able to predict the sensitivity of pemetrexed therapy in lung cancer, and that expression of *TS* mRNA and TS protein differed between squamous cell lung cancer or NSCLC and lung adenocarcinoma [5]. However, patients with similar histotype and disease stage might respond very differently to identical treatments, which suggests that TS genotypes contribute to these patients’ sensitivity to chemotherapeutic drugs. Therefore, clinical studies have begun to focus on developing personalized treatments based on both histotype and genotype to improve the efficacy of chemotherapy.

Indeed, it has been reported that the expression of *TS* was induced in various cancers, including gastric, colorectal, breast, and bladder cancers, compared with corresponding normal tissues [6,7]. Functionally, TS generates thymidine monophosphate (dTMP), which is subsequently phosphorylated to thymidine triphosphate for use in DNA synthesis and repair. *TS* is localized on chromosome 18, covering 16 kb, and with 7 exons that translates into a 72-kD protein and functions as a homodimer. A previous study showed that a 6-bp variation at 1494 bp of the *TS* 3'-UTR was associated with *TS* mRNA stability and reduced TS protein expression [8]. Other studies showed that this polymorphism also affected sensitivity to anti-cancer drugs and prognoses of breast and gastrointestinal cancers [6,9-11].

In the present study, we first detected expression of *TS* mRNA and TS protein in NSCLC tissue specimens, and then determined the genotype at the *TS* 3'-UTR at 1494del in 106 lung adenocarcinoma patients to investigate its association with sensitivity to pemetrexed.

## Methods

### Tissue specimens

A total of 160 chemotherapy-naive patients received surgery at the Department of Thoracic Surgery, First Affiliated Hospital of Liaoning Medical College between January 2006 and March 2010. All tumors were confirmed histologically as adenocarcinoma or squamous cell carcinoma of the lung. Representative fragments of fresh tumor and adjacent normal-appearing lung tissue were obtained and immediately snap frozen and stored in liquid nitrogen until used. The remaining tissue was fixed in formalin and embedded in paraffin. Our hospital review board, the Ethics Committee of First Affiliated Hospital of Liaoning Medical College, approved our study, and each patient or their legal guardian signed a consent form for participation in the study.

### Semi-quantitative reverse-transcription (RT) PCR

Total RNA was extracted from tissue specimens using Trizol (Tiangen Biotech, Beijing) in accordance with the manufacturer’s instructions. cDNA was transcribed from RNA samples using an iScript cDNA synthesis kit (BioRad, Hercules, CA). Primers for amplification of *TS* mRNA (NC_000018) were: 5’-TTGGACAGCCTGGGATTCTC-3’ (forward) and 5’-AGCTGGCGATGTTGAAAGG-3’ (reverse). Primers for β-actin were 5’-GCATCCACGAAACTACCTT-3’ (forward) and 5’-CTCGTCATACTCCTGCTT-3’ (reverse), which were synthesized by Aoke Biotech (Beijing). The PCR amplification was set to 94°C for 2 min, then 35 cycles of 94°C for 45 s, 58°C for 45 s, and 65°C for 90 s, and then 65°C for 10 min. PCR products were separated in a 1.5% agarose gel by electrophoresis at 90 V for one hour. β-actin mRNA was the internal control.

For semi-quantitative PCR, we first tested different cycles of PCR to amplify the *TS* mRNA (25–40 cycles) and chose 35 cycles for our study. After PCR products were resolved in a 1.5% gel, UV-light photos were taken and the density of the bands was quantified using Gel-pro Analyzer 4.5 software (Media Cybernetics, USA). The expression of *TS* mRNA was normalized as 47% of β-actin.

### Immunohistochemistry

Tissue sections were prepared, deparaffinized, and rehydrated in accordance with standard protocols. Endogenous peroxidase activity was quenched with 6% hydrogen peroxide in methanol for 30 min. Antigen retrieval was achieved by heating the tissue sections in a pressure cooker using 10 mM citrate buffer at pH 6. To block nonspecific binding, the sections were incubated with normal goat serum diluted in phosphate buffered saline (PBS) for 30 min. After washing with PBS, the sections were incubated with primary antibody overnight at 4°C followed by a 30-min incubation with horseradish peroxidase-conjugated secondary antibody at room temperature. The monoclonal mouse anti-human antibody against thymidylate synthase and a PV-6000 immunohistochemistry kit were purchased from Zhongshan (Beijing, China). The sections were then incubated in a 3,3’-diaminobenzidine chromogen solution and counterstained with Mayer’s hematoxylin. Negative controls were prepared as above, but without the primary antibody. Sections with known TS expression served as the positive controls. The intensity of TS expression was scored as 0, 1+, 2+, and 3+. A score ≥2+ was classified as positive for TS.

### Patients and treatment plan

One hundred and six patients were recruited from the Department of Oncology, First Affiliated Hospital of Liaoning Medical College between January 2006 and March 2010. Of the 160 consecutive chemotherapy-naive patients who received surgery at the Department of Thoracic Surgery, 119 cases were lung adenocarcinoma and 41 were lung squamous cell carcinoma. Thirteen patients from the 119 lung adenocarcinomas were lost to follow-up, resulting in 106 cases for data analysis.

The patients were histologically or cytologically confirmed as stage IIIB or IV lung adenocarcinoma and subjected to pemetrexed treatment. The patients had adequate organ functions and an Eastern Cooperative Oncology Group (ECOG) performance status of 0, 1, or 2. The hospital’s ethics review board approved the study protocol for pemetrexed therapy and all patients signed written informed consent before treatment.

Prior to pemetrexed infusion, the patients took 4 mg dexamethasone orally twice a day for three days. The patients also took 400 μg folic acid once a day, beginning 7 days before pemetrexed infusion and for 21 days after the final treatment. In addition, the patients were injected (intravenous) with 1000 μg vitamin B_12_ on the day before the first pemetrexed treatment and then once every three cycles of pemetrexed administration thereafter. The patients were given 500 μg/m^2^ pemetrexed through a single intravenous infusion that lasted more than 10 min, repeated once every 3 weeks.

Before entering the study, these patients underwent a physical examination and tumor measurement with computed tomography (CT) or magnetic resonance imaging (MRI). Overall survival (OS) after pemetrexed use was measured from the first date of pemetrexed therapy to the date of death or the last follow-up. A perspective clinical follow-up, including chest CT scan and telephone inquiry, was performed for all patients. Follow-up time was calculated from the end of chemotherapy until death, recurrence, metastasis, or the completion of the study in June 2011. Disease status was assessed according to the Response Evaluation Criteria in Solid Tumors [12]. Complete response (CR) or partial response (PR) was considered evidence of short-term effective treatment. A treatment that resulted in stable disease (SD) or progressive disease (PD) was judged ineffective. Long-term efficacy analysis included evaluations of OS and progression-free survival (PFS). PFS was defined as the time from the first date of pemetrexed therapy to the date of documented progression or death from any cause.

### Quantitative PCR (qPCR) detection of the TS gene 3’-UTR polymorphism

**Figure 1:**
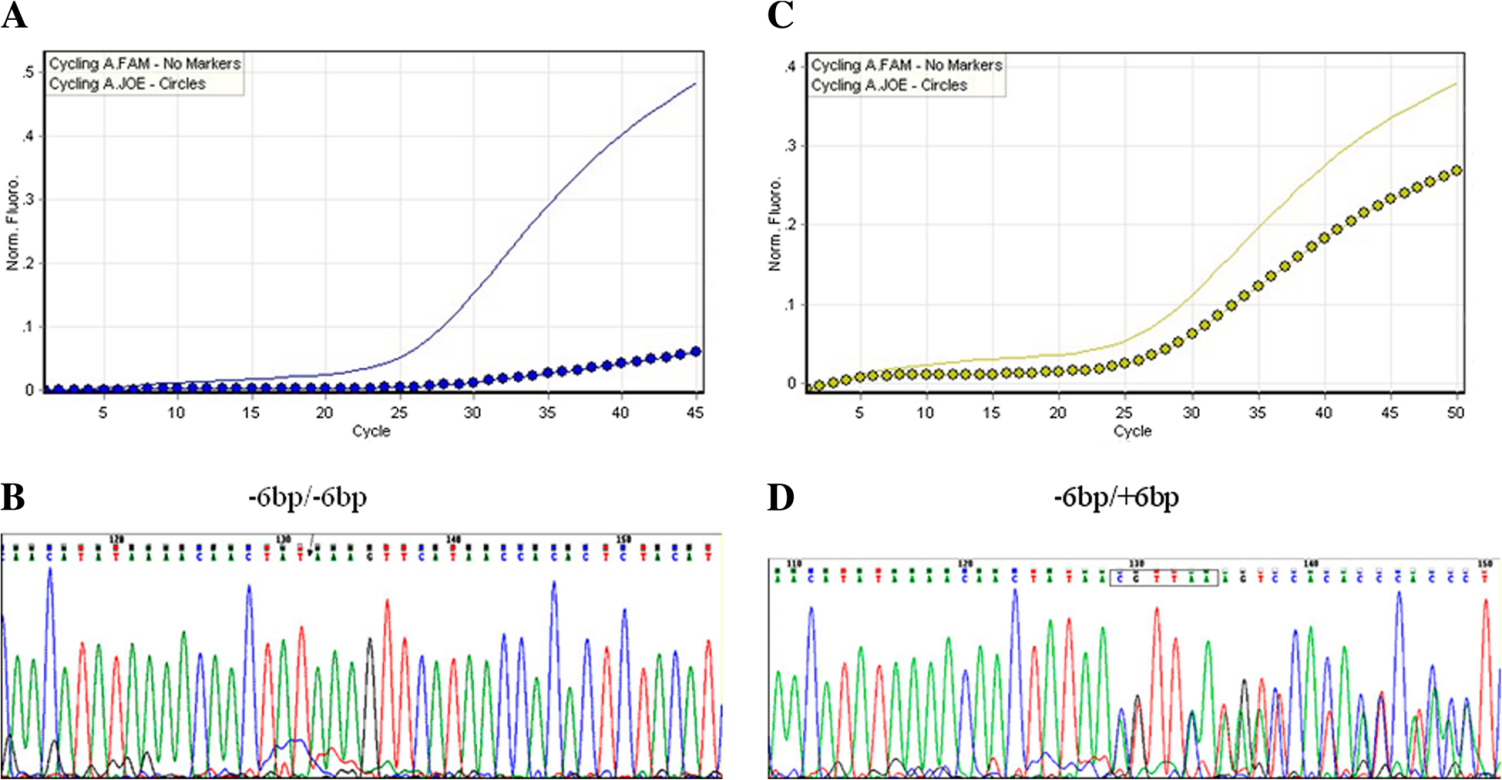
**Detection of *TS* gene 3’-UTR 6 bp polymorphism at 1494 bp.** Genomic DNA was extracted from blood from patients and subjected to qPCR analysis of the *TS* gene polymorphism and then to DNA sequencing analysis. **A** and **C**, qPCR amplification curves show *TS* 3’-UTR 1494 bp (−6 bp/-6 bp) and (−6 bp/+6 bp) polymorphisms. **B** and **D**, DNA sequencing data on *TS* 3’-UTR 1494 bp (−6 bp/-6 bp) and (−6 bp/+6 bp).

Genomic DNA was extracted from peripheral blood of the patients using the AxyPrep Mag Blood Genomic DNA kit (Axygen, Union City, CA) in accordance with the manufacturer’s instructions. To detect the *TS* gene 3’-UTR polymorphism at 1494 bp, the fragment containing the region was amplified using qPCR with primers rs34489327-FP (5’-GAGCTGAGTAACACCATCGA-3’), rs34489327-RP (5’-AGGAAGGAACTGAGC-AGATAA-3’), rs34489327-PJ (5’-FAM-TTATGAACTTTTTAAAGA-MGB-3’), and rs34489327-CR (5’-HEX-ATGAACTTTATAGTTGTT-MGB-3’) (AllGlo™). The PCR program was set at 95°C for 5 min, and then 45 cycles of 95°C 10 s and 55°C 40 s using a Rotor-Gene 3000 A (Corbett Research, Australia). Representative qPCR reaction curves of the *TS* 3’-UTR 1494 genotypes (−6 bp/-6 bp) and (−6 bp/+6 bp) are shown in Figure 1A and C. Three randomly selected PCR products from each genotype were subjected to DNA sequence analysis for confirmation of the *TS* 3’-UTR alterations.

### Statistical analyses

All statistical analyses were performed using SPSS 17.0 software (SPSS, Chicago, IL). For the assessing the association of TS expression with clinicopathological features, the probability (*P*) value was calculated using the chi-squared (χ^2^) test. *TS* mRNA expression is shown as mean ± standard deviation.

Grade correspondence analysis was performed using the non-parametric Spearman’s rank correlation coefficient. Expressions of *TS* mRNA and TS protein was analyzed with McNemmar and Spearman’s tests. The Kaplan-Meier method was used to estimate PFS and OS. Survival rates were compared with the log-rank test. Multivariate analysis of the independent prognostic factor for survival was performed using the Cox proportional hazard regression model with a 95% confidence interval (CI). A 5% or lower *P-value* was *considered statistically significant*.

## Results

### Increased expression of *TS* mRNA and TS protein in NSCLC

In this study, we first collected 160 clinically annotated NSCLC samples and corresponding normal tissues between January 2006 and March 2010. The baseline patient and disease-related characteristics are shown in Table 1.

**Table 1:** Association of TS protein expression with clinicopathological data from patients with NSCLC

**Characteristic**	**n**	**Positive expression of TS protein**	**Positivity(%)**	**χ**^**2**^	***P***
Age (years)					
< 60	72	45	63	1.1	0.29
≥ 60	88	62	70		
Gender					
Male	84	51	61	3.0	0.08
Female	76	56	74		
Disease stage					
IIIB	108	75	69	1.0	0.32
IV	52	32	62		
ECOG grade					
0	48	28	58	2.4	0.31
1	74	53	72		
2	38	26	68		
Histology					
Squamous cell carcinoma	41	34	83	6.4	0.01
Adenocarcinoma	119	73	61		

**Figure 2:**
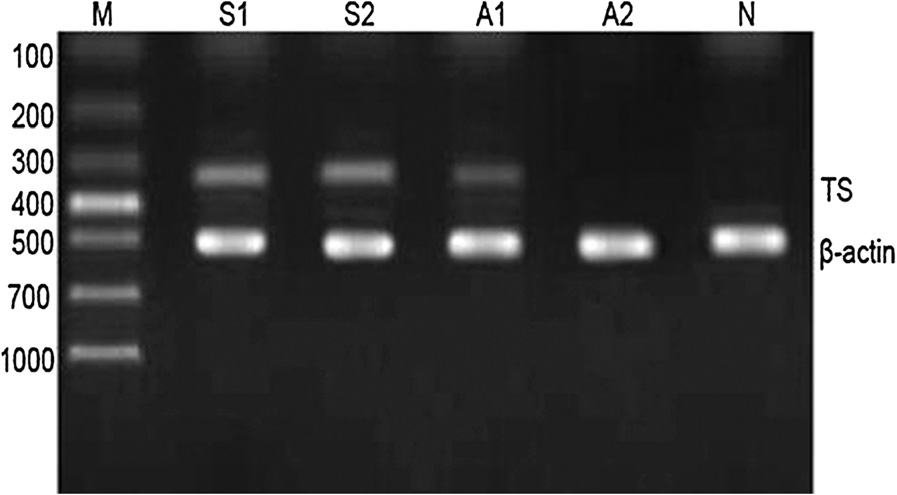
**Elevated expression of *TS* mRNA in NSCLC tissue specimens compared to normal tissues.** Surgical specimens were taken from patients with lung adenocarcinoma (A) or squamous cell carcinoma (S) and the corresponding normal mucosae (N) and subjected to semi-quantitative RT-PCR analysis of TS expression. The data are summarized as mean ± standard deviation relative to β-actin. The size of TS and β-actin PCR products was 337 bp and 500 bp, respectively. S (0.712 ± 0.183) compared with A (0.712 ± 0.183), P < 0.05.

*TS* mRNA was expressed in 73.13% (117/160) of NSCLC samples and 66.25% (92/160) of the normal tissue samples (Figure 2), a difference that is statistically significant (*χ*^*2*^*=*8.621*, P* = 0.003). Moreover, levels of *TS* mRNA were significantly higher in lung squamous cell carcinoma than in lung adenocarcinoma (0.71 ± 0.18 compared with 0.21 ± 0.23, *P* < 0.05).

**Figure 3:**
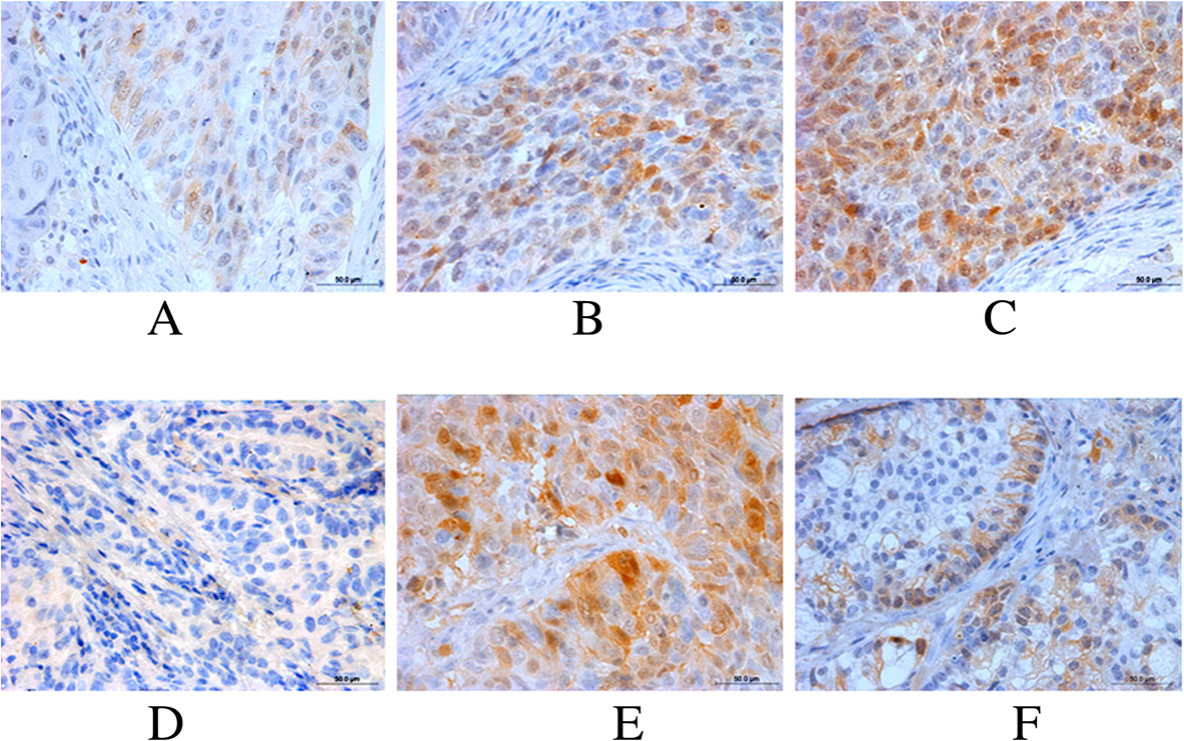
**Enhanced expression of TS protein in NSCLC compared with the corresponding normal tissues.** Paraffin sections from patients with NSCLC were prepared and stained with anti-TS antibody and then reviewed and scored under a microscope. **A-C**, Representative low, medium, and high levels of TS expression, respectively. **D**, Negative staining of normal tissues. **E**, strong expression of TS protein in lung squamous cell carcinoma. **F**, low level of TS expression in lung adenocarcinoma.

TS protein was expressed in 66.88% (107/160) of NSCLC tissues, which was significantly higher than that of normal tissues (57.5%, 79/160; *χ*^*2*^ *=* 10.066*, P* = 0.002). TS protein was mainly expressed in the nuclei, with some in the cytoplasm (Figure 3). In addition, 83% of lung squamous cell carcinoma showed positive TS staining, which was significantly higher than that of lung adenocarcinoma (61%; *P* = 0.01).

We then investigated the association between the expression of TS protein and the patients’ clinicopathological characteristics. We found that expression of TS protein was significantly associated with tumor histology (*P* < 0.05), but did not associate with other clinical features such as age, gender, disease stage, or ECOG grade (*P* > 0.05; Table 1). Expression of *TS* mRNA was positively correlated with protein expression as shown by Spearman’s test (r = 0.861, *P* = 0.000).

### *TS* gene 3’-UTR 6 bp polymorphism at 1494del associated with clinical outcome of NSCLC patients after pemetrexed treatment

We treated 106 patients with stage IIIB or IV lung adenocarcinoma for up to 6 cycles of pemetrexed (Table 2). The efficacy of pemetrexed treatment was assessed for all 106 patients.

**Table 2:** Association of pemetrexed treatment responses with clinicopathological data from lung adenocarcinoma patients

	**No. of patients**	**Efficacy**	**χ**^**2**^	***P *value**
		**CR** + **PR**		
Age (years)				
< 60	50	16	3.7	0.06
≥ 60	56	9		
Gender				
Male	62	10	4.6	0.03
Female	44	15		
Disease stage				
IIIB	78	24	5.6	0.02
IV	28	1		
ECOG grade				
0	36	13	5.0	0.08
1	54	10		
2	16	2		
Genotype				
−6 bp/-6 bp	59	19	5.5	0.02
−6 bp/+6 bp	47	6		
Expression of TS protein				
TS(+)	67	11	5.1	0.02
TS(−)	39	14		

Blood samples were collected from each patient and genotyped for the *TS* gene 3’-UTR polymorphism at 1494del 6 bp (Figure 1A and C). We found two genotypes of 3’-UTR 1494 bp in these patients, -6 bp/-6 bp and -6 bp/+6 bp. Three samples of each genotype were subjected to DNA sequencing for confirmation of the polymorphisms (Figure 1B and D). The data showed that 55.7% (59/106) of patients were (−6 bp/-6 bp), 44% (47/106) were (−6 bp/+6 bp), and 0% (0/106) were (+6 bp/+6 bp). The allelic distribution was in Hardy-Weinberg equilibrium (*χ*^2^ = 6.6, *P* = 0.83).

We then determined the association of these polymorphisms and clinicopathological data with sensitivity to pemetrexed, and short- and long-term efficacy of pemetrexed treatment, based on Response Evaluation Criteria in Solid Tumors (RECIST). Statistical analysis showed that gender, tumor stage, and the polymorphism of the *TS* 3’-UTR 1494del 6 bp were all prognostic factors for short-term efficacy of pemetrexed treatment (*P* < 0.05, Table 2). Of the (−6 bp/-6 bp) patients, 32% displayed a positive short-term response, whereas the responses of 13% of the (−6 bp/+6 bp) patients were also favorable. Furthermore, logistic regression analysis revealed that the difference in short-term efficacy between these 2 genotypes was statistically significant (*P* = 0.008), indicating that the *TS* 3’-UTR 1494del 6 bp polymorphism is an important prognostic predicator.

**Figure 4:**
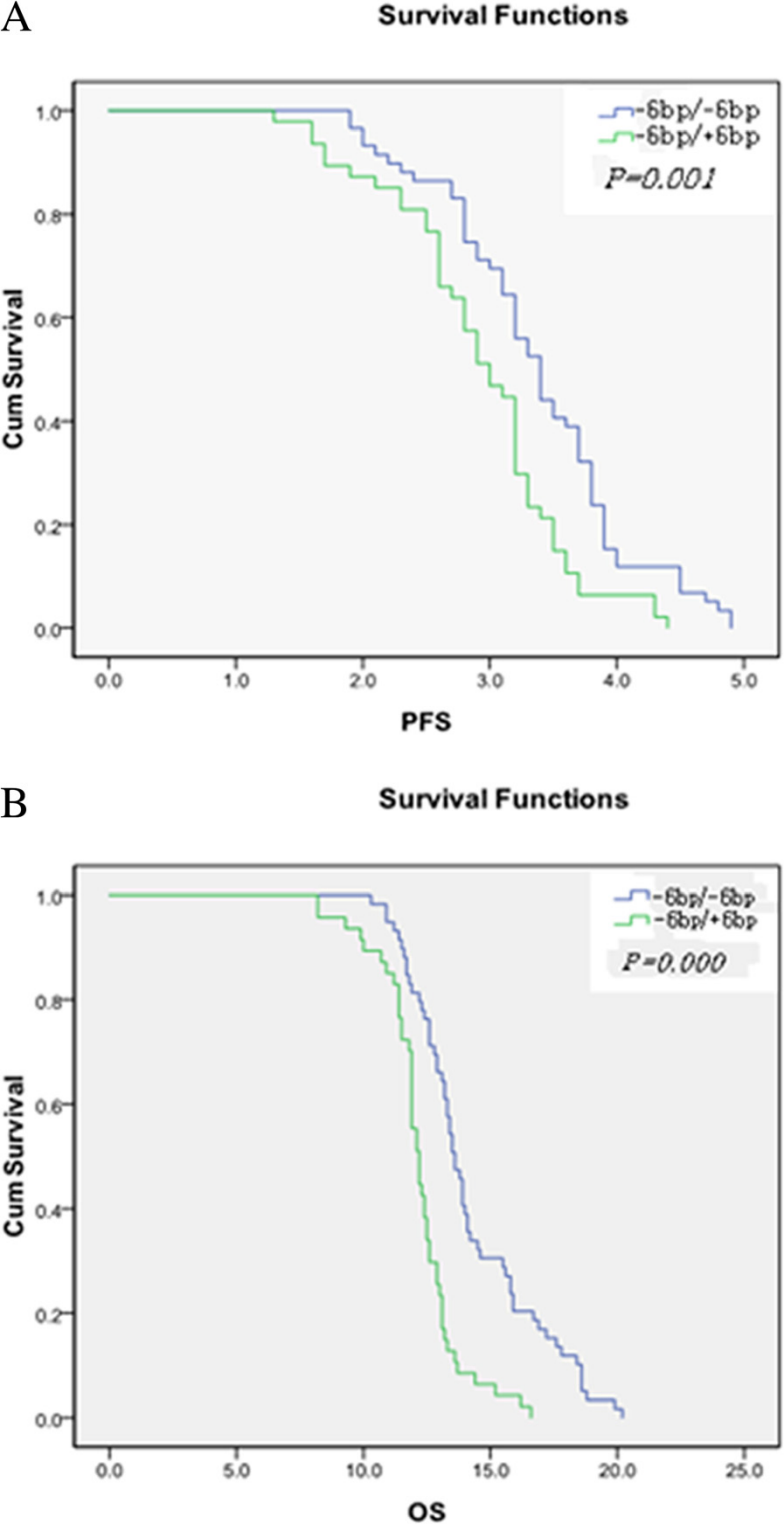
**Kaplan-Meier analysis of survival in lung adenocarcinoma and *TS* 3’-UTR 6 bp 1494del polymorphisms.** Kaplan-Meier plot of progression-free survival (PFS, **A**) and overall survival (OS, **B**) of the patients by genotypes (−6 bp/-6 bp) (blue line, n = 59) or (−6 bp/+6 bp) (green line, n = 47).

The Kaplan-Meier curves (Figure 4) revealed that after treatment with pemetrexed, the median PFSs of (−6 bp/-6 bp) and (−6 bp/+6 bp) patients were 3.4 and 2.9 months, respectively. Log-rank analysis revealed that patients with (−6 bp/-6 bp) had significantly better PFS than patients with (−6 bp/+6 bp) (χ^2^ = 12, *P* = 0.0010, Figure 4A). Moreover, the median OS of (−6 bp/-6 bp) and (−6 bp/+6 bp) patients were 14 and 12 months, respectively. Statistical analysis showed a significantly better OS for patients with the (−6 bp/-6 bp) genotype (*χ*^2^ = 27, *P* = 0.000, Figure 4B). Cox regression analysis showed that age, tumor stage, the type of polymorphism, and expression of TS protein of the *TS* 3’-UTR 1494del 6 bp significantly affected the OS of patients (*P* < 0.05), whereas gender and the ECOG score did not influence the survival significantly (Table 3).

**Table 3:** **Multivariate analysis of patients**’ **survival according to clinicopathological data and TS gene 3**’-**UTR 1494 bp polymorphisms**

	**Regression coefficient (b)**	**Standard errors**	**Wald**	***P***	**Exp (b)**	**95% CI**
Gender	−0.31	0.22	2.0	0.16	0.73	0.48-1.1
Age	0.61	0.22	7.5	0.01	1.8	2.0-2.8
Tumor stage	0.77	0.24	11	0.00	2.1	1.4-3.5
ECOG grade	−0.27	0.16	2.8	0.10	0.80	0.55-1.1
Genotype	0.92	0.27	12	0.01	2.5	1.5-4.3
Positive expression of TS protein	−0.60	0.28	4.6	0.03	0.55	0.32-1.0

### Association between adverse drug reactions and *TS* gene 3'-UTR 1494 bp

Adverse drug reactions of these patients to pemetrexed treatment were defined in accordance with the National Cancer Institute Common Toxicity Criteria (NCI-CTC) version 3.0 [13]. The most frequently occurring adverse drug reaction was bone-marrow inhibition, followed by gastroenterological discomfort and liver and kidney damage. However, no patients withdrew from treatment due to adverse drug reactions. Patients with the -6 bp/-6 bp genotype had more adverse drug reactions to the drug treatment than -6 bp/+6 bp patients, but the difference was not statistically significant (Table 4). Overall, the patients tolerated and complied with this drug treatment, making pemetrexed useful for controlling tumor progression and improving the quality of life of these advanced lung cancer patients.

**Table 4:** **Association between the adverse drug reactions and TS gene 3**'-**UTR 1494 bp**

	**Grade**	**Genotype** (**n** = **cases**)		**χ**^**2**^	***P***
		−/− (**n** = **59**)	−/+ (**n** = **47**)		
Leukopenia	I, II	22	16	0.057	0.483
	III, IV	0	1	1.241	0.449
Neutropenia	I, II	21	19	0.117	0.437
	III, IV	0	0	-	-
Thrombocytopenia	I, II	10	11	0.457	0.330
	III, IV	0	0	-	-
Nausea, Vomiting	I, II	9	10	0.447	0.337
	III, IV	0	1	1.241	0.449
Diarrhea	I, II	11	9	0.003	0.573
	III, IV	0	0	-	-
Constipation	I, II	14	10	0.057	0.498
	III, IV	0	0	-	-
Fatigue	I, II	15	10	0.155	0.435
	III, IV	0	0	-	-
Renal damage	I, II	0	0	-	-
	III, IV	0	0	-	-
Liver damage	I, II	2	1	0.143	0.590
	III, IV				

## Discussion

Our current study showed that TS is highly expressed in NSCLC and that polymorphisms of *TS* 3’-UTR 1494del 6 bp are associated with lung adenocarcinoma patients’ sensitivity to pemetrexed treatment. Otake et al. [14] was the first to show TS overexpression in NSCLC, i.e., 60.9% of 23 resected NSCLC samples displayed TS protein expression. Furthermore, they also performed the fluorodeoxyuridine-5'-monophosphate binding assay and found that TS enzymatic activity ranged from 1.8 to 56.9 pmol/g protein in NSCLC samples, indicating that TS may be involved in NSCLC tumorigenesis [14]. Nakagawa et al. [15] also found that NSCLC cell proliferation was associated with increased levels of TS expression, especially in lung adenocarcinoma cells. Our current study showing upregulated expression of *TS* mRNA and TS in NSCLC tissue specimens compared with normal tissues, confirmed the data of Otake et al. [14] and Nakagawa et al. [15]. The upregulated levels of TS expression in squamous cell lung carcinoma were associated with poor tumor differentiation, which is consistent with a study reported by Ceppi et al. [16].

However, Hashimoto et al. [17] showed that expression of *TS* mRNA was associated with lymph node metastasis, clinical stage, and tumor cell proliferation of lung adenocarcinoma. Although the data of the present study showed that TS expression in patients with lymph node metastasis and stage IIIA was higher than that of patients without lymph node metastasis and with stage I or II, the difference did not reach statistical significance.

Polymorphisms of the *TS* gene have been reported to correlate with clinical outcomes of chemotherapy. For example, Mandola et al. [10] showed that the *TS* 3’-UTR 1494del 6 bp polymorphism was correlated with low TS expression in colorectal cancer. Dotor et al. [9] reported that colon cancer patients with *TS* 3’-UTR 1494del 6 bp exhibited better response to 5-FU-based adjuvant therapy. A previous Chinese study [18] reported a similar result, i.e., *TS* 3’-UTR 1494del 6 bp was correlated with sensitivity to chemotherapy in advanced gastric cancer patients. However, another study reported that there was no association between this polymorphism and clinical outcome after chemotherapy [7]. These data indicate that polymorphisms of *TS* 3’-UTR 1494del 6 bp may be related to the response to particular chemotherapy agents or with different types of cancer. In our current study, we found an association between *TS* 3’-UTR 1494del 6 bp polymorphisms and outcome of pemetrexed treatments in lung adenocarcinoma patients.

A previous study reported that the +6 bp/1494 deletion polymorphism was more frequent in Caucasians (Los Angeles, CA), while the -6 bp/1494 was dominant in Chinese (Singapore) [10]. Gao et al. [19] found that Chinese patients with gastric cancer had 44.8% (−6 bp/-6 bp), 44.3% (−6 bp/+6 bp), and 7.6% (+6 bp/+6 bp) *TS* gene polymorphisms. In the present study, the short-term response to pemetrexed treatment was 32.20% and 12.77% in (−6 bp/-6 bp) and (−6 bp/+6 bp) patients, respectively, indicating that patients with the (−6 bp/-6 bp) polymorphism had a better response rate to pemetrexed treatment than those with the (−6 bp/+6 bp) polymorphism (HR: 4.382; 95% CI: 1.462 to 13.130; *P* = 0.008). A recent clinical trial of stage III NSCLC patients (known as the JMDB trial) with pemetrexed in combination with cisplatin found that the overall response rate was 27.1% [20]. While the current study supports the effectiveness of pemetrexed for NSCLC patients, the JMDB study did not include an analysis of *TS* gene 3’-UTR 1494 bp polymorphisms.

The current study found that the PFS and OS between patients with (−6 bp/-6 bp) and (−6 bp/+6 bp) were significantly different. The multivariate analysis showed that older age, advanced disease stage, and the *TS* 3’-UTR 1494 bp (−6 bp/+6 bp) genotype were prognostic for a poorer outcome. However, these data need to be confirmed in a large perspective clinical trial. In addition, with our current data it was not possible to analyze the association between the *TS* polymorphisms (−6 bp/-6 bp) and (−6 bp/+6 bp) and induced expression of TS in the NSCLC tissues available to us in this study. This is because we were unable to obtain any tissue specimens from pemetrexed-treated patients. Fortunately, a previous study did mechanistically demonstrate the link between them [21].

## Conclusions

This study showed that TS protein is highly expressed in NSCLC and that polymorphisms of *TS* 3’-UTR 1494del 6 bp are associated with sensitivity of lung adenocarcinoma patients to pemetrexed treatment. Our study suggests that *TS* gene polymorphisms should be further evaluated as prognostic markers for personalized chemotherapy in lung adenocarcinoma.

## Abbreviations

3’-UTR: 3'-untranslated region; CR: Complete response; CT: Computed tomography; dTMP: Thymidine monophosphate; ECOG: Eastern Cooperative Oncology Group; MRI: Magnetic resonance imaging; NSCLC: Non-small cell lung cancer; OS: Overall survival; PBS: Phosphate buffered saline; PD: Progressive disease; PFS: Progression-free survival; PR: Partial response; RECIST: Response Evaluation Criteria in Solid Tumors; SD: Stable disease; TS: Thymidylate synthase.

## Competing interests

The author(s) declare that they have no competing interests.

## Authors’ contributions

XW and YW carried out the molecular genetic studies, participated in the sequence alignment, and drafted the manuscript. YW carried out the immunoassays. JC and YW participated in the sequence alignment. MH conceived the study and participated in its design and coordination, performed the statistical analyses, and helped draft the manuscript. All authors read and approved the final manuscript.
